# A Good Woman Never Grows Old

**Published:** 1878-08

**Authors:** 


**A Good Woman Never Grows Old.**—Years may pass over her head, but if benevolence and virtue dwell in her heart, she is cheerful as when the spring of life opened to her view. When we look at a good woman we never think of her age; she looks charming as when the rose of youth first bloomed on her cheek. That rose has not faded yet; it will never fade. In her neighborhood she is the friend and benefactor. Who does not respect and love the woman who has passed her days in acts of kindness and mercy? We repeat, such a woman can never grow old. She will always be fresh and buoyant in spirits, and active in humble deeds of mercy and benevolence.

